# Nicotinic Acetylcholine Receptor Subtype Alpha-9 Mediates Triple-Negative Breast Cancers Based on a Spontaneous Pulmonary Metastasis Mouse Model

**DOI:** 10.3389/fncel.2017.00336

**Published:** 2017-11-03

**Authors:** Li-Chi Huang, Ching-Ling Lin, Jia-Zheng Qiu, Chun-Yu Lin, Kai-Wen Hsu, Ka-Wai Tam, Jung-Yu Lee, Jinn-Moon Yang, Chia-Hwa Lee

**Affiliations:** ^1^Department of Endocrinology, Cathay General Hospital, Taipei, Taiwan; ^2^School of Medical Laboratory Science and Biotechnology, College of Medical Science and Technology, Taipei Medical University, Taipei, Taiwan; ^3^Institute of Bioinformatics and Systems Biology, National Chiao Tung University, Hsinchu, Taiwan; ^4^Research Center for Tumor Medical Science, China Medical University, Taichung, Taiwan; ^5^Graduate Institute of Clinical Medicine, College of Medicine, Taipei Medical University, Taipei, Taiwan; ^6^Division of General Surgery, Department of Surgery, Shuang Ho Hospital, Taipei Medical University, Taipei, Taiwan; ^7^Department of Surgery, School of Medicine, College of Medicine, Taipei Medical University, Taipei, Taiwan; ^8^Department of Biological Science and Technology, National Chiao Tung University, Hsinchu, Taiwan; ^9^Department of Laboratory Medicine, Shuang Ho Hospital, Taipei Medical University, Taipei, Taiwan; ^10^Comprehensive Cancer Center of Taipei Medical University, Taipei, Taiwan

**Keywords:** triple-negative breast cancer, cancer metastasis, gene editing, α9-nAChR, epithelial–mesenchymal transition

## Abstract

Triple-negative breast cancer (TNBC) subtype is associated with poor prognosis and a high risk of recurrence-related death in women. Despite the aggressiveness of TNBCs, targeted TNBC therapy is not yet available in the clinic. To overcome this challenge, we generated highly metastatic TNBC cells (LM) derived from metastasized lung cells via a serial spontaneous pulmonary metastasis animal model to identify targetable molecules for attenuating the progression of TNBC metastasis. Gene analysis of primary tumor (P), first-round (1LM) and second-round (2LM) metastasized lung cells revealed that mesenchymal-related genes were significantly expressed in LM cells, especially in 2LM cells. Interestingly, α9-nAChR gene expression was also dramatically induced in LM cells, confirming our previous finding that α9-nAChR plays important roles in receptor-mediated carcinogenic signals in human breast cancer development. Using α9-nAChR as a biomarker, we transfected 2LM cells with CRISPR/Cas9 lentivirus targeting the α9-nAChR genomic region (2LM-α9-nAChR-null), showing that mesenchymal markers and the migration and invasion abilities of 2LM cells were significantly attenuated in 2LM-α9-nAChR-null cells both *in vitro* and *in vivo*. In addition, the high efficiency of editing the α9-nAChR gene using a CRISPR/Cas9 lentivirus was demonstrated by gene sequencing, genomic indel frequency and protein expression analyses. Collectively, these results confirmed those of our previous study that advanced-stage breast tumors are associated with substantially higher levels of α9-nAChR gene expression, indicating that α9-nAChR expression is essential for mediating TNBC metastasis during cancer development and may potentially act as a biomarker for targeted therapy in clinical investigations.

## Introduction

In 1851, Claude Bernard first discovered that nicotine directly activates muscle tissue, and nicotine was later proven to neurally stimulate common receptors through neurotransmitter release. Over the past two decades, clinical investigations have revealed that nicotinic acetylcholine receptors (nAChRs) play essential roles in mediating anti-nociception (Marubio et al., 1999), cognitive performance (Lange-Asschenfeldt et al., 2016), and oncogenic characteristics in lung, colon, and breast cancers (Dasgupta et al., 2006; Wei et al., 2009; Lee et al., 2010, 2011). Among all nAChRs, the homopentameric form of a7-nAChR and the heteropentameric form of a4b2-nAChR are the two most investigated nAChRs, as they are the focus of studies exploring the downstream signaling pathways and biofunctional properties of nAChRs. Upon stimulation, nicotine reacts as acetylcholine binds with a higher affinity to a4b2-nAChR than to a7-nAChR (Barik and Wonnacott, 2006). However, a7-nAChR is reportedly a powerful regulator of responses that stimulate cancer cells (Schuller and Orloff, 1998), mediating basal cell proliferation and differentiation pathways in lung and airway remodeling during bronchopulmonary diseases, providing strong evidence of a7-nAChR expression in the development, repair, and carcinogenic progression of lung cancer (Maouche et al., 2009).

In addition to a7-nAChR, the homopentameric form of α9-nAChR has been reported to play important roles in promoting cancer cell proliferation (Lee et al., 2010), angiogenesis (Cooke, 2007), cancer metastasis (Wei et al., 2009), and apoptosis suppression (Paliwal et al., 2010) during carcinogenesis in response to tumor microenvironments. A novel approach to block oncogenic signals via nAChRs has been used in preclinical and clinical trials to discover targeting agents (including agonists and antagonists) in selective nAChR subtypes, especially a4b2-, a7-, and α9-nAChR, which provide receptor activation with less desensitization (Wu et al., 2011) in cells. Some of the potential agents have even attenuated the effects of nAChR in clinical therapy (Holladay et al., 1997; Matsubayashi et al., 2004; Wang et al., 2005).

The homopentameric structure of α9-nAChR, initially discovered in hair cells of the inner ear, is involved in the developmental processes of adult auditory function and synaptic transmission in efferent signings (Glowatzki and Fuchs, 2000). Moreover, many biofunctional characteristics of α9-nAChR have been identified in keratinocyte adhesion (Nguyen et al., 2000), immune responses (Luebke et al., 2005), neuropathic pain (Vincler et al., 2006), and even breast epithelial cancer formation (Lee et al., 2010). Accordingly, the correlation between α9-nAChR expression in tumors and prognosis outcomes in 276 breast cancer patients was evaluated, showing that 67.3% of breast tumor tissues expressed higher levels of α9-nAChR than adjacent normal tissues (Lee et al., 2010). In addition, the authors discovered that higher α9-nAChR expression was found more often in advanced-stage (stages 3 and 4) breast cancer tissues than in early stages (stages 1 and 2), and this occurred more often in smokers than in passive smokers or non-smokers. This study suggest that α9-nAChR expression may initiate strong signals, thus enhancing breast cancer metastasis during tumor development.

Cancer metastasis is a crucial process in tumor development, whereby cancer cells spread from the primary tumor to distant tissues. Accordingly, more than 90% of cancer-associated deaths are strongly linked to distant metastasis (Spano et al., 2012). Cancer metastasis is composed of several essential steps, including cancer cell proliferation, migration, invasion, adhesion, and angiogenesis. The progression of cancer cell invasion contains the most crucial biological activities, as it involves the intravasation of blood vessels, the circulation of tumor cells in the bloodstream, and the extravasation of distant tissues. Several factors have been found to play important roles in regulating this process, including growth factor stimulation and extracellular matrix component production, terminating and reassembling in actin cytoskeleton for cell shape and motility remodeling (Burridge and Chrzanowska-Wodnicka, 1996). However, the current metastasis animal model used most often via mouse tail-vein injection initiates from circulating tumor cells and lacks crucial steps, such as cancer cell proliferation, angiogenesis, cell migration and vessel intravasation. Thus, to overcome these limitations, we established highly metastatic triple-negative breast cancer (TNBC) cells via spontaneous pulmonary metastasis in a severe combined immunodeficiency (SCID) mouse model to identify novel metastatic genes with the ability to home-in on and thrive in lung metastatic lesions. Using the CRISPR/Cas9 gene editing strategy, we verified the important and essential roles of the molecules both *in vitro* and *in vivo*. From this study, we envision that our animal model will promote the development of therapeutic strategies to prevent TNBC metastases and improve TNBC patient survival in the near future.

## Materials and Methods

### Cell Lines and Culture Conditions

Human mammary gland epithelial adenocarcinoma cell line MDA-MB-231 and human kidney epithelial Phoenix-ECO cells were purchased from the American Tissue Culture Collection (ATCC, Manassas, VA, United States) and maintained in Dulbecco’s Modified Eagle Medium: Nutrient Mixture F-12 (DMEM/F-12) Media (Gibco, United States). The cells were incubated with 10% (v/v) fetal bovine serum (FBS, Biological Industries, Israel). The supplement of 100 units/ml penicillin and 100 mg/ml streptomycin were used and cultured in a 37°C incubator with 5.0% CO_2_. The medium was replaced every 2 days, and when cells reached 80% confluence, they were passaged using 0.25% trypsin/EDTA (Gibco, United States).

### Transfection and Cell Line Selection

MDA-MB-231 cells were transfected with pcDNA3 plasmids expressing the firefly luciferase gene (the gene sequences were originally from *luc4.1*; Chris Contag, Stanford University, Stanford, CA, United States), as described previously (Tu et al., 2017). Briefly, 5 × 10^6^ MDA-MB-231 cells were washed twice with phosphate buffered saline (PBS) and mixed with 10 mg of plasmid. Two pulses were applied for 20 ms under 1.2 kV on the pipette-type MicroPorator MP-100 (Digital Bio, Seoul, Korea). The stable cells were selected 48 h later with G418 (6 mg/mL). Bioluminescent derivatives of the MDA-MB-231 cells were used for further *in vitro* and *in vivo* studies.

### Animal Experiments

Four-week-old severe combined immunodeficient (SCID) female mice were purchased from the National Science Council Animal Center (Taipei, Taiwan) and housed in micro-isolator cages at the Laboratory Animal Center in the National Defense Medical Center (Taipei, Taiwan). This study was carried out in strict accordance with the recommendations in the *Guide for the Care and Use of Laboratory Animals* from the National Institutes of Health. All surgeries were performed under isoflurane anesthesia and all efforts were made to minimize suffering. During the experiment, no stress or abnormal behaviors due to tumor bearing were observed in the mice. The health status of the animals was monitored once daily by a qualified veterinarian. Food and water were replaced every 2 days.

### Orthotropic Lung Metastasis Animal Model

Our orthotropic tumor model used immune-competent SCID mice (4 weeks old) to mimic cancer in humans. Five mice were anesthetized with 2% isoflurane, and the mammary pads of each mouse were implanted with 5 × 10^6^ luciferase-expressing MDA-MB-231 cells. Throughout the study, all mice were kept in an environmentally controlled room maintained between 21–24°C and 43–65% relative humidity. During the experiment, all animals underwent bioluminescent imaging every week for lung metastasis observation.

### Bioluminescent (IVIS) Imaging

Bioluminescent imaging was performed with a highly sensitive, cooled CCD camera mounted in a light-tight specimen box (In Vivo Imaging System—IVIS; Xenogen). Before imaging was taken, the mice were i.p.-injected with D-luciferin (200 mg/kg) 15 min in advance. The animals were placed on the warmed stage inside the camera box and received continuous exposure to 2.5% isoflurane to sustain sedation during imaging. Every group of mice was imaged for 1, 5, 10, and 30 s. The light emitted from the mice was detected by the IVIS camera system, integrated, digitized, and displayed. Regions of interest from the displayed images were identified and quantified as total photon counts or photons using Living Image^®^software 4.0 (Caliper, Alameda, CA, United States).

### Flow Cytometry Analysis

The MDA-MB-231 derived cells were collected and stored in a 75% alcohol-PBS solution for flow cytometry analysis. The forward scatter (FSC) measurement was conducted by FACSCalibur (BD Biosciences).

### Real-Time Quantitative Polymerase Chain Reaction (Q-PCR)

The human metastatic-related gene primers are listed in the Supplementary Table S1. All oligo primers were synthesized by Genomics BioSci and Tech (Taipei, Taiwan). A LightCycler thermocycler (Roche Molecular Biochemicals, Mannheim, Germany) was used for Q-PCR analysis. One microliter of the sample and master-mix was first denatured for 10 min at 95°C and then incubated during 40 cycles (denaturation at 95°C for 5 s; annealing at 60°C for 5 s; elongation at 72°C for 10 s) to detect fluorescent intensity. All of the PCR samples underwent melting curve analysis for non-specific PCR product detection. The gene expression results from the Q-PCR analysis were normalized with human β-glucuronidase (GUS) expression as an internal control using the built-in Roche LightCycler Software, Version 4.

### Absolute Q-PCR

To generate the absolute quantitative standard curve for Q-PCR analysis, we used the PCR product of the mouse GUS gene and cloned it with the TA cloning vector (*pTA*^®^Easy Cloning Kit), which was purchased from Genomics BioSci and Tech (Taipei, Taiwan). After following the steps of gene sequencing, *E. coli* amplification, plasmid purification, and determination of molecular weight, the copied GUS genes were calculated and diluted by 10^8^ to 10^2^ per ml. Each copied gene was measured for accuracy and liner correlation.

### Protein Extraction, Western Blotting, and Antibodies

For western-blot analysis, MDA-MB-231 derived cells were washed once with ice-cold PBS and lysed with radioimmunoprecipitation assay (RIPA) lysis buffer containing protease inhibitors. For *in vivo* experiments, the tumor tissues were cut into small pieces and lysed with RIPA lysis buffer containing protease inhibitors. The cell and tumor samples were homogenized three times at setting 3 (18,000 rpm) on ice using a PRO 200 homogenizer (PRO Scientific Inc., Monroe, CT, United States). Fifty micrograms of protein from each sample was resolved by sodium dodecyl sulfate polyacrylamide gel electrophoresis (SDS-PAGE) and transferred to a nitrocellulose membrane. The anti-Snail (sc-271977), anti-a-SMA (sc-53015), and anti-GAPDH (sc-32233) antibodies were purchased from Santa Cruz Biotechnology (Santa Cruz, CA, United States), and the anti-Slug (#9585), anti-Vimentin (#5741), anti-*N*-cadherin (#13116), and anti-*E*-cadherin (#3195) antibodies were purchased from Cell Signaling Technology (Danvers, MA, United States). The secondary anti-mouse and rabbit antibodies were purchased from Santa Cruz Biotechnology. All of the primary antibodies were used at 1000 dilution, overnight hybridization, followed by a 1-h incubation with a 1:4000 dilution of the secondary antibodies.

### Lentiviral Production and Cell Transduction

Lentiviral particles were produced by the transient transfection of Phoenix-ECO cells (CRL-3214) using TransIT^®^-LT1 Reagent (Mirus Bio LLC, Madison, WI, United States). Guide oligonucleotides were phosphorylated, annealed, and cloned into the BsmBI site of the lentiCRISPR v2 vector (Addgene, 52961, kindly provided by Feng Zhang) according to the Zhang laboratory protocol (Shalem et al., 2014) (F. Zhang lab, MIT, Cambridge, MA, United States). All of the constructs were verified by sequencing, and viral constructs were co-transfected with pMD2.G (Addgene plasmid 12259) and psPAX2 (Addgene plasmid 12260) (both kindly provided by Didier Trono, EPFL, Lausanne, Switzerland). Lentiviral particles were collected at 36 and 72 h before being concentrated with Lenti-X Concentrator^®^(Clontech, Mountain View, CA, United States). The lentivirus concentration for each gene was quantified by Q-PCR. MDA-MB-231 cells were infected in a 6-cm dish, with each well containing 1 × 10^6^ cells and an MOI (multiplicity of infection) = 3. Two days after transfection, the medium was replaced with 2.5 mg/ml puromycin for 2 days of cell selection. Lentivirus-transfected cells were recovered 2 days before the experiments. The use of biohazards and restricted materials involved in this study were followed by “Safety Guidelines for Biosafety Level 1 to Level 3 Laboratory.” The protocol was approved by the institutional biosafety committee at Taipei Medical University, Taipei, Taiwan.

### Sequencing of Single Guide RNA (sgRNA) Target Sites

Genomic DNA was extracted and PCR amplified the α9-nAChR gene region using the following primers: forward 5′-..GAGAGGCTGGTGACACGTAA..-3′ and reverse 5′-..TTTGTAGAAAGCTTCCTTTTCCCG..-3′. The PCR products were purified using a PCR clean-up Purification Kit and were sequenced by the Sanger method using forward PCR primers. The editing efficiency of the sgRNAs and the potential induced mutations were assessed using Tracking of Indels by Decomposition (TIDE) software^1^ (Netherlands Cancer Institute), which only required two Sanger sequencing runs from wild-type cells and mutated cells.

### RNA-Guided Engineered Nuclease-Restriction Fragment Length Polymorphism (RGEN-RFLP) Assay

PCR products (∼100 ng per assay) were incubated for 30 min at 37°C with the Cas9 protein [New England Biolabs (NEB), Beverly, MA, United States] and α9-nAChR sgRNA in 10 μl of NEB buffer 3. After cleavage, RNase A (2 μg) was added, and the reaction mixture was incubated for 15 min at 37°C to remove RNA. Next, proteinase K (2 μg) was added, and the reaction mixture was incubated for 15 min at 58°C to remove the Cas9 protein. The products were resolved on 2% agarose gels and visualized with ethidium bromide (EtBr) staining.

### Detecting Cell Migration by the Wound-Healing Assay and the Boyden Chamber

For the wound-healing assay, MDA-MB-231 cells were seeded in culture inserts (ibidi, Planegg/Martinsried, Germany) and cultured until the cells reached confluence. The culture inserts were removed, and the cells were photographed at 0, 12, and 24 h. The cells were then fixed in paraformaldehyde for 15 min at room temperature and stained with Hoechst 33258 for 15 min at room temperature. Healing areas were compared to the initial wound areas at various times, and migrated cells derived from MDA-MB-231 cells were counted using ImagePro Plus 6.0 software.

For detecting cell migration using the Boyden chamber, Matrigel was coated onto 0.8 μM invasion inserts (Corning, Corning, NY, United States), which were placed in a 37°C incubator and allowed to solidify for 1 h. MDA-MB-231-derived cells (2.5 × 10^4^) were seeded into the upper chamber in serum-free media. A medium containing 10% FBS was placed in the lower chamber, and the cells were incubated at 37°C in a humidified atmosphere with 95% air and 5% CO_2_. After 48 h, the inserts were washed in PBS, and the cells were fixed with 4% paraformaldehyde for 15 min at room temperature and then stained with crystal violet (0.5% of PBS) for 15 min at room temperature. Five fields on each insert were imaged by a cell microscope and quantified using ImagePro Plus 6.0 software.

### Immunofluorescent (IF) Staining

To investigate lung metastasis in the animal model, IF and HE (hematoxylin and eosin) staining assays were performed on poly-L-lysine-coated slides. The slides were incubated with FITC-labeled anti-Vimentin antibodies for 2 h at room temperature, washed twice with phosphate-buffered saline, and incubated with secondary antibodies for an additional 1 h in a moist chamber at room temperature. The slides were stained with Hoechst 33258 for nuclei location and examined with a Leica DMI3000 B Fluorescence Microscopy Imaging System (Leica Microsystems, Wetzlar, Germany). To enhance the color contrast of Vimentin and nuclei location, The FITC signal was displayed with green color, and nuclei location was displayed with red color.

### Statistical Methods

All data were expressed as mean ± SD, and student *t*-test analysis was performed for the pairwise samples. All statistical comparisons were performed using SigmaPlot graphing software (San Jose, CA, United States) and Statistical Package for the Social Sciences v.13 (SPSS, Chicago, IL, United States). A *P*-value < 0.05 was considered statistically significant, and all statistical tests were two-sided.

## Results

### Highly Metastatic Breast Cancer Selection via an *in Vivo* Spontaneous Metastasis Mouse Model

**FIGURE 1:**
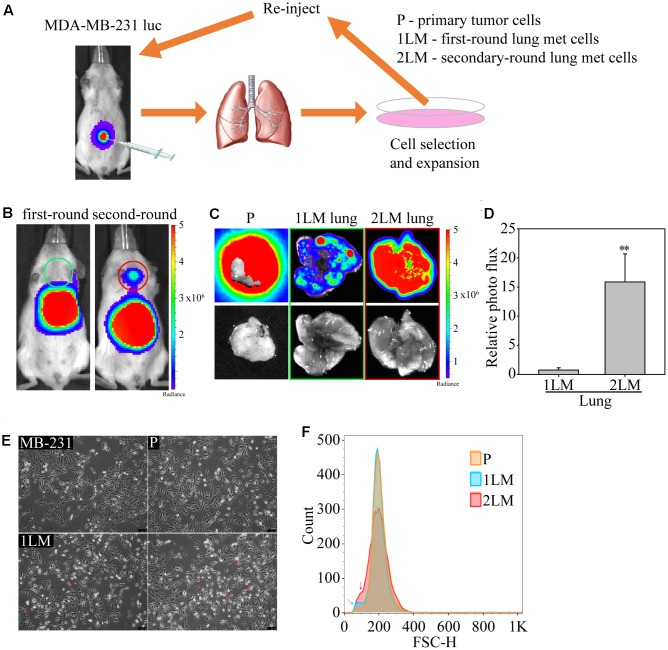
*In vivo* selection of highly metastatic breast cancer cells. **(A)** Schematic representation of the *in vivo* selection process by an advanced spontaneous metastasis mouse model. Luciferase-labeled MDA-MB-231 (MB-231) cells were inoculated subcutaneously into SCID mice, which were subjected to bioluminescence analysis 4 weeks after injection. Primary tumor cells (P) and metastatic tumor cells (1LM) from first-round xenograft mice were isolated from tumors and lung tissues, respectively. Metastatic tumor cells were reinoculated after expansion in cell culture. Metastatic tumor cells (2LM) isolated from the second round were expanded in cell culture, and metastasized cells were selected from the expanded cell culture with G418. **(B)** Bioluminescence images of mice inoculated with first- and second-round metastatic tumors. **(C)** Bioluminescence (upper panel) and bright field (bottom panel) images of lung tissues inoculated with primary tumors, first-round tumors, and second-round tumors. **(D)** Comparative luciferase activity of lung tissues inoculated with first- and second-round cells, *n* = 3–5. Data are presented as the mean and standard deviation errors. **(E)** Cell morphologies of derived MB-231, P, 1LM, and 2LM cancer cells. Smaller cells are indicated by red arrow headed. **(F)** The average metastatic breast cancer cell size was evaluated by flow cytometry. Cell measurement is presented as the cell count. P cells are presented in orange, whereas 1LM (blue arrow headed) and 2LM cells (red arrow headed) are presented in blue and red, respectively.

In this study, we established a highly metastatic TNBC cell line derived from metastasized lung cells (lung mets, LM) via a serial spontaneous pulmonary metastasis animal model to evaluate significant gene alterations associated with TNBC metastasis. The MDA-MB-231 cells were highly invasive and originally isolated from the pleural effusion of a patient whose disseminated disease relapsed several years after removal of the primary tumor (Cailleau et al., 1978). MDA-MB-231 cells are known to metastasize to multiple sites, including the brain, lungs, and bones, when inoculated into immunodeficient mice (Kang et al., 2003; Minn et al., 2005; Bos et al., 2009). To begin the cell derivation, luciferase-labeled MDA-MB-231 cells (MB-231) were expanded in culture and inoculated into first-round recipient mice. After 16 weeks, the mice were sacrificed, and their tumor cells were dissociated from the tumors and termed “primary tumor cells (P)” (**Figure 1A**). To further examine the metastatic potential of lung cells, we dissociated tumor cells from the lung tissue of TNBC-carrying mice, termed “first-round lung mets-derived cells (1LM).” 1LM cells were expanded in culture and reinoculated into second-round recipient mice to harvest metastatic lung cells. Consequently, metastasized lung cells from the 1LM tumor xenograft model were established as the “second-round lung mets-derived cells (2LM).” Both 1LM and 2LM cells were inoculated into mouse mammary fat pads via orthotopic injection. Second-round lung metastasis was clearly detectable in the mice by bioluminescence imaging 12 weeks after the inoculation of 1LM cells and appeared in most of the inoculated animals (**Figure 1B**). To validate the invasion ability of 1LM cells, P tumor cells and the lungs of mice inoculated with 1LM and 2LM cells from the lung metastasis xenograft tumor model were assayed for bioluminescence activity (**Figure 1C**). The data clearly showed that 2LM cells had luciferase activity 15% higher than that of 1LM cells (**Figure 1D**). Notably, cell morphology imaging analysis showed that 2LM cells were smaller and rounder than 1LM cells (red arrow headed), indicating that the 2LM cells may have lost their desmosome and adherens junction properties (**Figure 1E**). This finding was also confirmed by flow cytometry analysis, showing that 2LM cells were smaller in size than 1LM cells (blue arrow headed) and P cells (red arrow headed) (**Figure 1F**).

### Highly Metastatic TNBC-Enhanced Epithelial–Mesenchymal Transition (EMT)-Related Gene Expression Profile

**FIGURE 2:**
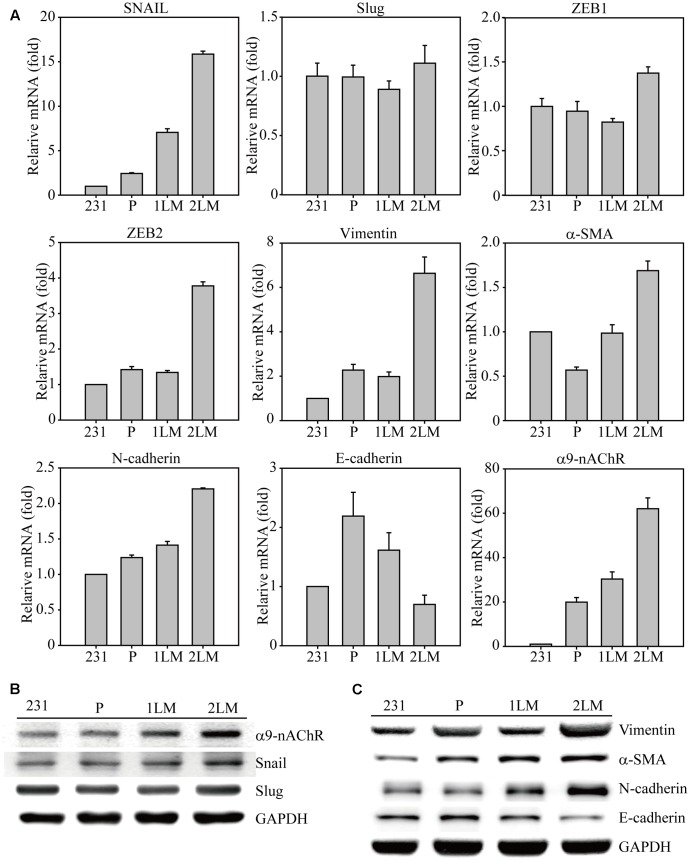
Epithelial–mesenchymal transition (EMT)-mediated gene regulation in highly metastatic breast cancer cells. **(A)** The cDNAs of MB-231, P, 1LM, and 2LM cancer cells were analyzed for EMT-related gene expression by real-time qualitative PCR (Q-PCR). The EMT-related genes included transcription factors (Snail, Slug, ZEB1, ZEB2), cytoskeleton proteins (Vimentin, a-SMA), cell adhesion proteins (*N*-cadherin, *E*-cadherin), and signal membrane receptors (α9-nAChRs). The cell lysates from four individual cell types were collected and analyzed for protein expression by western blot. The protein alterations were categorized as **(B)** cell signal transduction or **(C)** cell cytoskeleton behavior. Data are presented as the mean and standard deviation errors.

To identify significant gene alterations during TNBC development, we evaluated metastatic gene expression profiles in the metastatic-derived MB-231, P, 1LM, and 2LM cancer cells (**Figure 2A**). Gene expression of the transcription factor Snail was highly associated with metastatic ability and was 15-fold higher in 2LM cells than in MB-231 cells. However, Slug gene expression remained unchanged during TNBC development. The transcription factors ZEB1 and ZEB2 were shown to play important roles in the EMT process, whereas only ZEB2 was highly expressed in 2LM cells. In cytoskeleton, Vimentin and α-SMA are two of the most investigated cell structural genes during mesenchymal transition. Here, we found that both genes were predominantly expressed in 2LM cells compared to their expression in the other derived cell lines, indicating the unique malignant properties of Vimentin and α-SMA in 2LM cells. Cadherins are known to mediate cell adhesion and migration in relation to malignancy. In this study, we demonstrated that *N*-cadherin was highly associated with the increased metastatic ability of 2LM cells, whereas *E*-cadherin was negatively associated with metastatic ability.

In our previous study, α9-nAChR overexpression was discovered in 276 breast tumor regions compared to that surrounding normal breast epithelial cells, implying the malignancy role of α9-nAChR during breast cancer progression. Interestingly, a significant induction of α9-nAChR gene expression was closely correlated with metastatic ability in this animal model, as α9-nAChR gene expression was increased 30-fold in 1LM cells and 60-fold in 2LM cells compared with that in MB-231 cells. This observation confirmed our finding that α9-nAChR expression in breast tumor tissues is significantly higher in advanced stages (late-stage) in the clinical setting, indicating that α9-nAChR plays a strong metastatic role during breast tumorigenesis.

Furthermore, metastatic proteins in cell lysates from the four cancer-derived cell lines were assayed by western blot. The increased expression levels of α9-nAChR and Snail were strongly correlated with metastasis ability (2LM > 1LM > P), whereas Slug expression remained unchanged (**Figure 2B**). In addition, the cytoskeleton proteins Vimentin, a-SMA, and *N*-cadherin were highly expressed in 1LM- and 2LM-derived cells, especially in 2LM cells. The protein expression of *E*-cadherin, a negative regulator of cell adhesion, was gradually decreased in LM cells, wherein glyceraldehyde 3-phosphate dehydrogenase (GAPDH) served as the internal control.

Overexpressing α9-nAChR in normal breast epithelial cells subjected to long-term nicotine exposure has been shown to trigger cell transformation into precancerous cell types, which demonstrates the characteristics of cancer colony formation in soft agar and tumor formation in nude mice. To understand the metastatic roles of α9-nAChR via nicotine activation, we stimulated MDA-MB-231 cells with nicotine dose-dependently for 6 and 24 h (**Supplementary Figure S1**). Nicotine significantly induced a-SMA and α9-nAChR expression at concentrations of 5–10 mM, whereas *E*-cadherin expression was dramatically decreased at the same nicotine concentrations. In addition, Slug expression was also induced by nicotine treatment at 6 h. In summary, nicotine-induced TNBC mesenchymal marker expression and transcription factor activation were consistent with lung metastasis-induced gene expression. These data explain the observations from our previous study that α9-nAChR was profoundly expressed in the later stages (3 and 4) of breast cancer in smoking patients. Interestingly, most passive smokers and non-smokers diagnosed with early stage (0 and 1) breast cancer expressed lower levels of α9-nAChR, suggesting that nicotine may promote breast oncogenesis during cancer development via α9-nAChR expression.

### α9-nAChR Genome Editing Verified by Gene Sequencing

**FIGURE 3:**
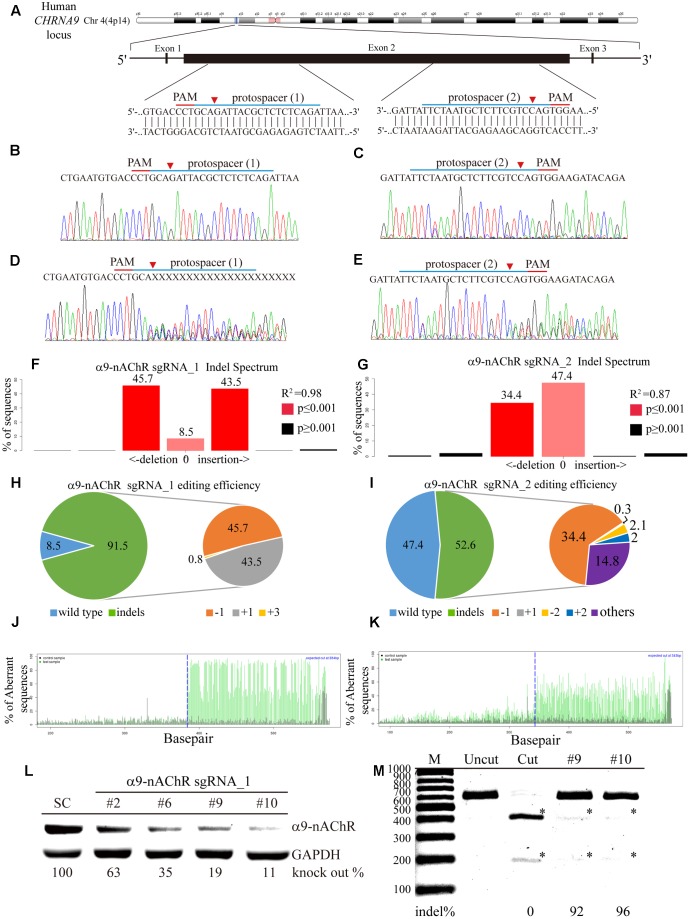
Genome editing assay based on α9-nAChR gene sequencing. **(A)** Schematic representation of the α9-nAChR DNA locus and two protospacer sequences (blue underline) for editing. The arrowhead indicates the expected Cas9 cleavage site. The protospacer adjacent motif (PAM, red underline) is the motif required for Cas9 nuclease activity. Sanger sequencing of the α9-nAChR DNA region was performed in 2LM cells. Scrambled SC sgRNA and α9-nAChR gene editing were accomplished by lentiviral delivery with a multiplicity of infection (MOI) = 5. Post transfection, DNA from virus-infected cells was purified and Sanger-sequenced for the α9-nAChR exon2 DNA locus, and 2LM cells expressing SC sgRNA were used as the control. **(B,C)** had wild-type sequences, while cells expressing **(D)** α9-nAChR sgRNA_1 and **(E)** α9-nAChR sgRNA_2 showed a mixture of sequences around the expected Cas9 cleavage point. TIDE decomposition algorithm analysis of the α9-nAChR gene-edited sequence (indel –insertion and deletion) in 2LM cells showed high editing efficiency at the expected cleavage point by **(F)** α9-nAChR sgRNA_1 and **(G)** α9-nAChR sgRNA_2 delivered via lentivirus. The commonly sequenced variants for CRISPR-targeted α9-nAChR transgenes in 2LM cells are shown. The pie charts show the indel percentages of the α9-nAChR gene edited by **(H)** α9-nAChR sgRNA_1 and **(I)** α9-nAChR sgRNA_2. The gene editing efficiency of the two sgRNAs are presented in green (left panel), while –1 and +1 indels are presented in orange and gray, respectively. The panels illustrate the aberrant sequence signal in the scrambled (black), **(J)** α9-nAChR_sgRNA1, and **(K)** α9-nAChR sgRNA_2 (green) cell pools and the expected cleavage site (vertical dotted line). Several α9-nAChR sgRNA_1- and α9-nAChR sgRNA_2-null MDA-MB-231 cell clones were isolated and expanded in culture. The selected cell lysates from each α9-nAChR sgRNA_1 null clone were assessed for **(L)** α9-nAChR protein expression by western blotting and **(M)** α9-nAChR gene editing by the RGEN-RFLP assay.

Next, we investigated the generalizability of CRISPR/Cas9 genome editing in breast cancer cells by targeting selected protospacers on the *CHRNA9* DNA locus (**Figure 3A**). To promote delivery efficiency, two custom-designed sgRNAs for the *CHRNA9* gene were cloned into the lentiCRISPR v2 plasmid to generate lentiviruses for gene editing purpose. Individual α9-nAChR sgRNA_1 and sgRNA_2 lentiviral infections were performed in 2LM cells, and scrambled sgRNAs were transferred for parental comparison. Post-infection, Sanger sequencing was used to verify indel (insertion or deletion) mutations at the expected locations in all CRISPR-Cas9 assays, and no changes were observed at locations targeted by the scrambled sgRNAs (**Figures 3B,C**). As expected, with both α9-nAChR sgRNA_1 and α9-nAChR sgRNA_2 viral infection, mixed *CHRNA9* sequences were located at the predicted genome cleavage sites (**Figures 3D,E**, arrowhead). Furthermore, tracking of indels by decomposition (TIDE) analysis identified α9-nAChR sgRNA_1 as the most efficient sgRNA among those tested, with 91.5% of the cell pool being edited (**Figure 3F**), whereas α9-nAChR sgRNA_2 achieved only a 52.6% gene editing efficiency (**Figure 3G**). The most frequently predicted mutations of the α9-nAChR sgRNA_1 cell pool were 1-bp deletions (45.7%) and 1-bp insertions (43.5%) (**Figure 3H**), whereas the frequently predicted mutations of the α9-nAChR sgRNA_2 cell pool were 1-bp deletions (34.4%) and other mutations (14.8%) (**Figure 3I**). Likewise, the algorithm for both α9-nAChR sgRNAs predicted the same patterns of genome repair, which included mutations mainly at the cleavage points (**Figures 3J,K**).

To confirm efficient editing of the *CHRNA9* gene by lentiviral CRISPR/Cas9, α9-nAChR protein expression was assessed by western blot. A total of 12 null clones from the α9-nAChR sgRNA_1 cell pool and two null clones from the α9-nAChR sgRNA_2 cell pool were obtained (**Supplementary Figure S2**). Notably, strong α9-nAChR protein expression was detected in the scramble (SC) cells, while null clones 2, 6, 9, and 10 from the α9-nAChR sgRNA_1 cell pool demonstrated weaker protein expression, especially null clones 9 and 11, which expressed α9-nAChR at only 19 and 11% of that of SC cells, respectively (**Figure 3L**). We also applied CRISPR/Cas9-derived RGEN-RFLP analysis to quantify α9-nAChR gene editing efficiency (**Figure 3M**). Genomic DNA purified from null clones 9 and 11 of the α9-nAChR sgRNA_1 cell pool were subjected to PCR amplification, and the PCR products of SC cells were subjected to RGEN-RFLP genotyping with (cut) or without (uncut) α9-nAChR sgRNA_1 (**Figure 3K**). Consistently, cleaved DNA obtained from clones 9 and 11 using the RGEN-RFLP assay was very weakly digested, yielding indels on the α9-nAChR gene of 92% and 96%, respectively. Thus, α9-nAChR DNA sequences from clones 9 and 11 were highly edited and could no longer be recognized by α9-nAChR sgRNA_1 or digested by the Cas9 protein.

### Migration and Invasion Properties of α9-nAChR-Null and Highly Metastatic TNBC Cells

**FIGURE 4:**
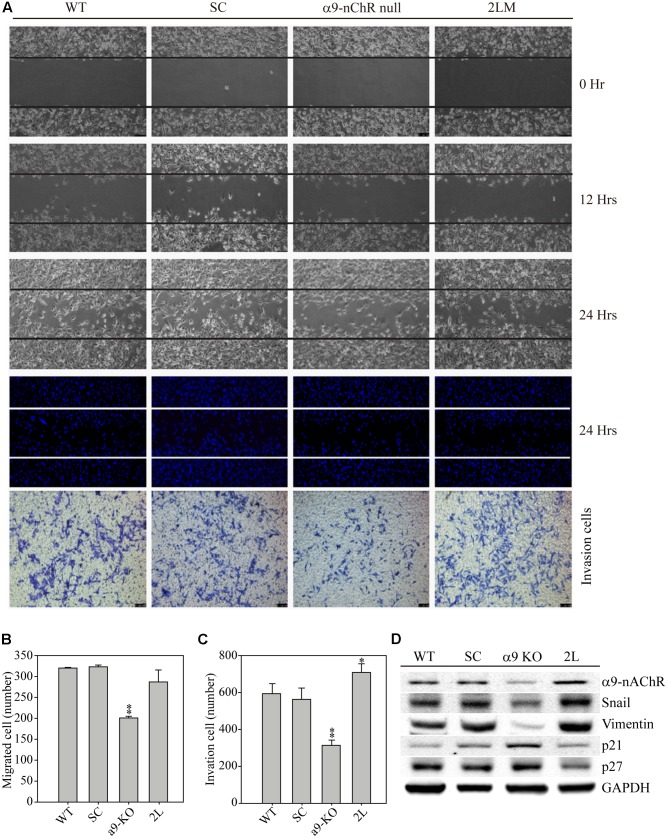
Cell migration and invasion abilities of α9-nAChR-null and 2LM TNBC cells. **(A)** The migration and invasion abilities of MB-231, SC, α9-nAChR-null, and 2LM cells were measured by the ibidi wound-healing chamber and the Boyden chamber with Matrigel. The migration photos were acquired at 0, 12, and 24 h after the culture chambers were removed. The cells were stained with Hoechst and counted to assess their migration ability. The invaded cells were stained with crystal violet 24 h after cell seeding. **(B)** Migration and **(C)** invasion cell counts of four types of breast cancer cells in a 24-h observation period. The values are presented as the means and standard deviation errors. Data were analyzed with Student’s *t*-test; all *P*-values were two-sided. *P*-values less than 0.05 are indicated with an asterisk, and *P*-values less than 0.01 are indicated with two asterisks. **(D)** EMT metastatic protein expression in the four types of breast cancer cells. GAPDH expression served as the loading control.

We next compared the migration and invasion properties of MB-231, SC, α9-nAChR-null, and 2LM cells. Four types of MDA-MB-231-derived cells were seeded 12 h prior to the assay in migration chambers. The following day, the chambers from each cell were removed, and images were recorded at 0, 12, and 24 h. The images clearly showed the suppressed migration ability of α9-nAChR-null cells at 12 and 24 h, whereas the other three cell types had higher migration abilities (**Figure 4A**). By contrast, 2LM cells were found to be more invasive than the parental MB-231 cells and SC cells, while α9-nAChR-null cells had the lowest invasion ability. Counting the number of migrated cells by Hoechst staining at the 24 h time point showed that α9-nAChR-null cells migrated the least (200 cells/image) of all the cell types (300 cells/image) (**Figure 4B**). Likewise, counting the number of invaded cells in the Boyden chamber by crystal violet staining at the 24 h time point showed that α9-nAChR-null cells migrated the least (300 cells/image), whereas 2LM cells exhibited the best migration ability (over 700 cells/image), superior to that of the MB-231 and SC cells (**Figure 4C**).

Next, we used the four cancer cell types to investigate whether α9-nAChR expression induces metastatic protein alterations (**Figure 4D**). Protein analysis clearly showed that α9-nAChR was highly expressed in 2LM cells and weakly expressed in α9-nAChR-null cells. In addition, the metastatic proteins Slug and Vimentin were also strongly expressed in 2LM cells and weakly expressed in α9-nAChR-null cells. On the other hand, the tumor suppressors p21 and p27 were highly induced in α9-nAChR-null cells and significantly reduced in 2LM cells, indicating that α9-nAChR regulates cancer cell migration and invasion by manipulating the expression of metastatic and tumor-suppressive proteins during TNBC development.

### 2LM and α9-nAChR-Null Cells in the *in Vivo* Metastasis Model

**FIGURE 5:**
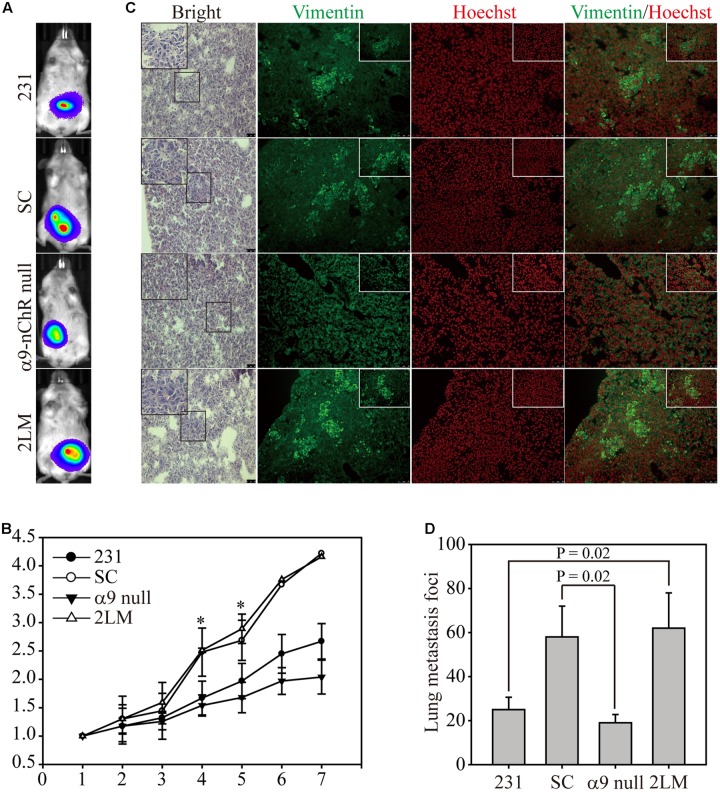
2LM and α9-nAChR-null cells in the *in vivo* metastasis model. **(A)** Luciferase-labeled MB-231, SC, α9-nAChR-null, and 2LM cells were inoculated into SCID mice subcutaneously. The mice underwent weekly bioluminescence analysis for 3 weeks after injection. The bioluminescence images from the four types of tumors at 7 weeks are shown. **(B)** Growth of the four tumor types was determined by their bioluminescent activity. The tumor size for each mouse was based on the size fold change from week 1. **(C)** The lung tissue slides from each group were stained with H&E or IF with Vimentin (green) or Hoechst (red). Scale bar, 50 μm. The small photos were acquired by a high-power lens. Scale bar, 25 μm. **(D)** Metastatic tumor foci from all mice were counted by a low-power lens. The values are presented as the means and standard deviation errors. Data were analyzed with Student’s *t*-test; all P-values were two-sided. *P*-value less than 0.05 are indicated with an asterisk.

Next, we determined whether cancer cells expressing α9-nAChR to different extents could affect lung metastasis in an animal model. Experimentally, 5x10^6^ cells of each type were injected orthotopically into mice, and tumor growth was monitored and visualized by bioluminescence to detect distant metastases (**Figure 5A**). Seven weeks post-measurement, growth of SC and 2LM tumors was significantly higher than that of parental MB-231 tumors, whereas α9-nAChR-null tumors exhibited the least growth. This indicates that the α9-nAChR protein plays a role in tumorigenesis *in vivo* (**Figure 5B**). After the mice were sacrificed, lungs from each mouse were sliced and stained with hematoxylin and eosin (H&E) or immunofluorescence (IF) for Vimentin (green) or Hoechst (red) (**Figure 5C**). In H&E-stained lungs, pathological images of cancerous cells were largely observed in lungs from the 2LM and SC groups, whereas lungs from the α9-nAChR-null group exhibited no pathological changes. In the IF-stained lungs, lungs from the 2LM and SC groups exhibited high Vimentin expression, while the lungs from the α9-nAChR-null group showed low Vimentin expression. Using a low-power lens, we calculated the tumor foci of all lung slides and found that 2LM tumors induced significantly more lung metastatic nodules (*p* = 0.02) than MB-231 tumors, indicating the rapidity and sensitivity of the spontaneous pulmonary metastasis mouse model for evaluating TNBC invasion ability *in vivo* (**Figure 5D**). Likewise, α9-nAChR-null tumors exhibited significantly fewer lung metastatic nodules (*p* = 0.02) than SC tumors, suggesting that targeting α9-nAChR will be a convincing strategy for suppressing TNBC tumorigenesis.

## Discussion

Injecting cancer cells into immunosuppressed mouse models, including nude and NOD SCID mice, via the tail-vein is often used to assess the metastatic capability of human breast cancer cell lines derived from tumors. However, the lack of reliable metastasis *in vivo* models has markedly hampered investigations of breast cancer metastatic processes, especially for TNBC. In a previous study, MDA-MB-231 cells were injected into the mammary fat pads of BALB/c nude mice, and no lung metastasis was found in five of the mice tested (Minn et al., 2005). “Trained cells” obtained through tail-vein injection or harvested from lung lesions could repeatedly metastasize from the mammary fat pad, but success was achieved in less than 50% of the mice. While our TNBC-based spontaneous pulmonary metastasis animal model shares a similar strategy of using “trained cells” initiated from primary tumor formation in orthotopic mammary fat pads, metastasis was observed in nearly 100% of the mice in our model using bioluminescence imaging, providing a robust model for the study of human breast cancer metastasis in mice. In addition, our results show that interaction in the primary tumor microenvironment is important for generating metastatic cancer cells. Thus, using highly metastatic cancer cells to identify key molecules that determine metastatic ability should be the first priority when researching TNBC.

Epithelial–mesenchymal transition is thought to contribute to tumor progression and is directly correlated with tumor aggressiveness and poor clinical outcomes (Cano et al., 2000). Thus far, transcriptional regulation of the cadherin family has been considered a key event during EMT. Human *N*-cadherin and *E*-cadherin are two of the most investigated cell adhesion molecules, and E-box elements of the *E*-cadherin promoter are required for direct binding with Snail (Hennig et al., 1995), Slug (Cano et al., 2000), Zeb1 (Hajra et al., 2002), and Zeb2 proteins to suppress *E*-cadherin transcriptional activity. Accordingly, high Slug expression has been found in a variety of tumors (Alves et al., 2009), including TNBC (Martin et al., 2005; Storci et al., 2008; Lehmann et al., 2011), which has been correlated with reduced *E*-cadherin expression, high cancer staging, lymph node micro-metastasis and poor patient survival outcomes (Bailey et al., 2012). In the current study, overexpression of only the Snail and ZEB2 genes was detected in LM cells, allowing the transcriptional activation of Vimentin, a-SMA and N-cadherin gene expression, whereas *E*-cadherin gene expression was inhibited. By contrast, the transcription factors Slug and ZEB1 are likely not involved in TNBC-related lung metastasis events. Interestingly, expression of the EMT regulator Twist was also unaltered in 1LM and 2LM cells, and the TWIST-Slug-ZEB1 metastatic correlation appeared to be biologically relevant in the invasion of cancer cells but not MDA-MB-231 cells.

nAChRs are well known to stimulate genes related to cancer cell proliferation and survival *in vitro* and *in vivo* (Schaal and Chellappan, 2014). Thus, many studies have focused on the effects of nicotine exposure mimicked by cigarette smoking on nAChR signaling machinery (Egleton et al., 2008; Grando, 2014). Accumulating evidence has suggested that either the PI3K/Akt or MAPK signaling pathways regulate cancer cell invasion and migration (Tsurutani et al., 2005; Shi et al., 2015a). This regulation may be due to the induction of actin filament remodeling by the activation signal to improve cell migration and matrix metalloproteinase (MMP) secretion, thus enhancing the invasion ability of breast cancer cells (Shin et al., 2016). Our previous study demonstrated that nicotine significantly induced Akt and Erk protein phosphorylation via α9-nAChR activation in both ER-positive and TNBC breast cancer cells (Lee et al., 2011). Therefore, the fact that α9-nAChR mediated signal transduction relates to our current finding that migration and invasion were dramatically reduced in α9-nAChR-null cells, whereas 2LM cells exhibited significantly enhanced migration and invasion abilities. These data provide strong evidence that α9-nAChR expression determines the metastatic ability of TNBC and that LM cells could serve as a robust animal model for assessing drugs relevant to anti-cancer- or anti-metastasis for TNBC therapy.

Recently, CRISPR-Cas9 genome editing has become a promising strategy for discovering therapeutic targets for cancer and other diseases (Shi et al., 2015b). Despite the broad interest in CRISPR-Cas9 genome editing, recognizing that off-target cleavages and unwanted protein translocations are potential hazards in CRISPR/Cas9 research and biotechnology due to the unspecific recognition of non-target sequences is critical (Yee, 2016). The major concern of off-target effects is that gene-edited chromosomal rearrangements may accidently disable tumor-suppressor genes or activate oncogenes, which could cause cellular toxicity (Brunet et al., 2009). In our CRISPR/Cas9 assay, no off-target sequences were observed in any of the edited pools of cells based on Sanger sequencing, demonstrating that CRISPR/Cas9 gene editing was highly specific, which was also illustrated in a previous whole-genome sequencing study (Veres et al., 2014).

In this study, we illustrated that the RGEN-RFLP assay is a very reliable and precise genotyping detection method. Combining Sanger sequencing-based mutational analysis (TIDE), we could measure gene editing efficacy (indel and substitution frequency) and whether the predicted genome-edited sites were cleaved using frame-shift DNA sequence curves. Unlike the traditional T7 endonuclease I and Surveyor gene editing measurement assays that can detect only 50% of gene-edited sequences, quantitative RGEN-RFLP analysis can detect 100% of these sequences. This advantage results in a better method for detecting indels when comparing DNA editing efficiency. In addition, according to our experiment, RGEN-RFLP indel detection was consistently correlated with target protein expression and Sanger sequencing confirmation via TIDE analysis. This shows that results from RGEN-RFLP and TIDE gene editing analyses are extremely reliable and promising, providing an appropriate solution when antibodies are insufficient for protein confirmation.

Although tobacco avoidance is still the best strategy for cancer prevention, the emerging understanding that nAChRs can mediate the carcinogenic mechanism in tumor progression provides the basis for a novel therapeutic biomarker for cancer prevention and/or cancer treatment in the clinical setting. The past concern was that nAChR antagonists or their inhibitors that inactivate receptors may be problematic because nAChRs regulate numerous vital cell and organ functions. In fact, current evidence has shown that α9-nAChR antagonists may be successful blockers as anti-cancer drugs with no cytotoxicity to normal control cells.

## Conclusion

Our *in vivo* spontaneous metastasis mouse model has contributed to the ability to test invasion-preventive drugs and identify novel metastasis-related genes during TNBC tumorigenesis. In this study, α9-nAChR was shown to impact a key molecular function in the lung-distant metastasis of MDA-MB-231 cells. Using CRISPR/Cas9 gene editing, we generated α9-nAChR-null cells from 2LM cells and found dramatic attenuation effects on cancer migration and invasion both *in vivo* and *in vitro*. Results from this study suggest that targeting α9-nAChR genes or proteins may be beneficial for patients affected by the aggressive malignancy of TNBC.

## Author Contributions

L-CH, C-LL, and K-WT provided the clinical advice for this study. J-ZQ performed the Q-PCR western blot analysis. C-YL, K-WH, and J-YL helped design the CRISPR protospacers and advised on the virus production and infection procedures. C-HL, L-CH, and C-LL helped interpret the results and gene editing efficiency analysis. J-MY provided bioinformatics advice on spontaneous pulmonary metastasis mouse model. C-HL designed all of the experiments and wrote the paper in conjunction with the other authors.

## Conflict of Interest Statement

The authors declare that the research was conducted in the absence of any commercial or financial relationships that could be construed as a potential conflict of interest.

## Footnotes

1 https://tide-calculator.nki.nl

## Supplementary Material

**FIGURE S1 |** MB-231 cells were exposed to dose-dependent nicotine treatment for six or 24 h. The cell lysates were washed with PBS and analyzed for protein expression by western blot.

**FIGURE S2 |** To confirm efficient editing of the *CHRNA9* gene by CRISPR-Cas9, α9-nAChR protein expression was assessed by western blot. A total of 12 null clones from the α9-nAChR sgRNA_1 cell pool and two null clones from the α9-nAChR sgRNA_2 cell pool were obtained.
