# Supplementary figures and images for: Nicotinic Acetylcholine Receptor Subtype Alpha-9 Mediates Triple-Negative Breast Cancers Based on a Spontaneous Pulmonary Metastasis Mouse Model
Source: Front Cell Neurosci. 2017 Nov 3;11:336. doi: 10.3389/fncel.2017.00336 (PMC5675882; doi:10.3389/fncel.2017.00336)

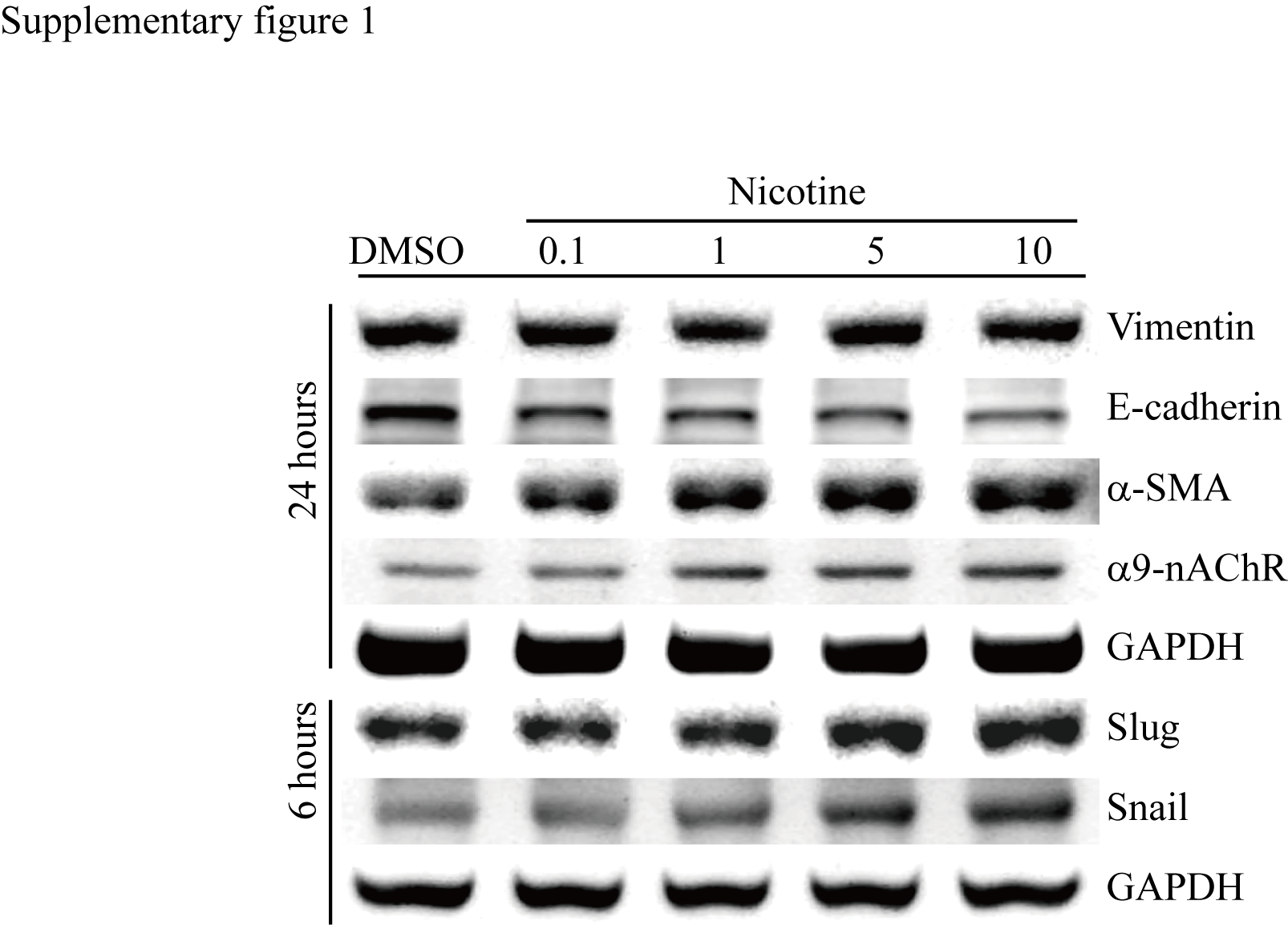

Supplement: Supplementary file 1 [file Image_1.TIF]

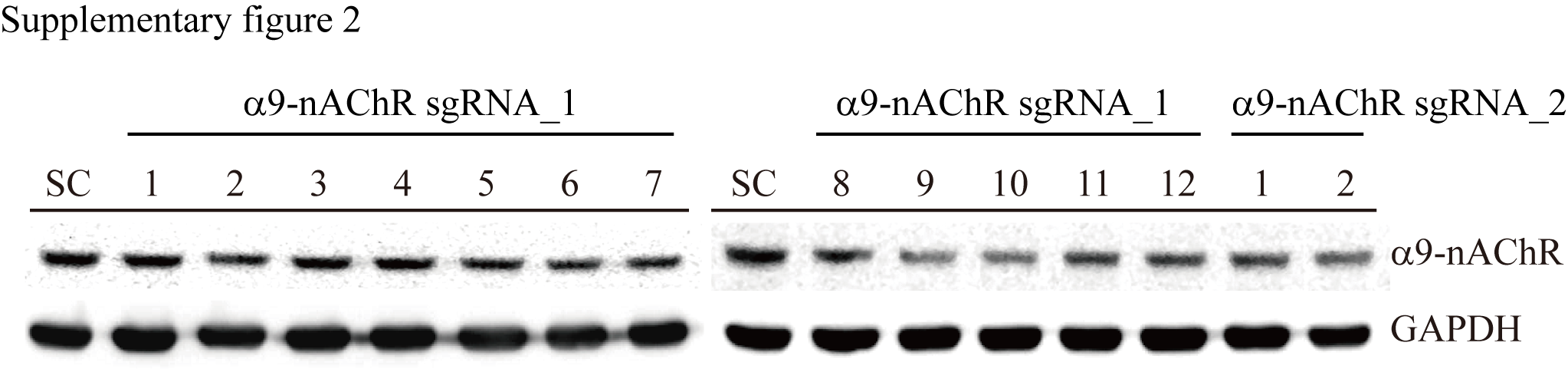

Supplement: Supplementary file 2 [file Image_2.TIF]
