# Supplementary material for: Nicotinic Acetylcholine Receptor Subtype Alpha-9 Mediates Triple-Negative Breast Cancers Based on a Spontaneous Pulmonary Metastasis Mouse Model
Source: Front Cell Neurosci. 2017 Nov 3;11:336. doi: 10.3389/fncel.2017.00336 (PMC5675882; doi:10.3389/fncel.2017.00336)
Supplement: Supplementary file 3 [file Table_1.DOCX]

**Supplementary table**

List of all QPCR primers and their sequences

| Primer name | | Sequence | Product (bp) |
| --- | --- | --- | --- |
| GUS | Forward | 5' - AAACAGCCCGTTTACTTGAG | 166 |
|  | Reverse | 5' - AGTGTTCC TGCTAGAATAGATG |  |
| α9-nAChR | Forward | 5' - CAGGGTCTTGTTTGTCTATGAT | 174 |
|  | Reverse | 5' - AGGTCGTTCTTTAAGCGT |  |
| SNAI1 | Forward | 5' - CCCAATCGGAAGCCTAAC | 158 |
|  | Reverse | 5' - AGGACAGAGTCCCAGATGA |  |
| ZEB2 | Forward | 5' - AGAGAGGAAGAGGAAGATGAAATA | 154 |
|  | Reverse | 5' - TGTTAATTGCGGTCTGGAT |  |
| Vimentin | Forward | 5' - ATTGAGATTGCCACCTACAG | 157 |
|  | Reverse | 5' - CCGTCTTAATCAGAAGTGTCC |  |
| α-SMA | Forward | 5' - ACTGAGCGTGGCTATTC | 152 |
|  | Reverse | 5' - TCAGGCAACTCGTAACTCTT |  |
| N-cadherin | Forward | 5' - ATGACAATCCTCCAGAGTTTAC | 152 |
|  | Reverse | 5' - ATCTCCGCCACTGATTC |  |
| E-cadherin | Forward | 5' - GCAGAACTAACACACGGG | 159 |
|  | Reverse | 5' - GGTGGTCACTTGGTCTTTATT |  |
